# Twenty years of newborn screening for congenital adrenal hyperplasia and congenital primary hypothyroidism – experiences from the DGKED/AQUAPE study group for quality improvement in Germany

**DOI:** 10.1515/medgen-2022-2114

**Published:** 2022-05-07

**Authors:** Johanna Hammersen, Markus Bettendorf, Walter Bonfig, Eckhard Schönau, Katharina Warncke, Alexander J. Eckert, Susanne Fricke-Otto, Katja Palm, Reinhard W. Holl, Joachim Woelfle

**Affiliations:** Department of Paediatrics, University Hospital Erlangen, Erlangen, Germany; Division of Paediatric Endocrinology and Diabetes, Department of Paediatrics, University Hospital Heidelberg, Heidelberg, Germany; Department of Paediatrics, Klinikum Wels-Grieskirchen, Wels, Austria; Children’s Hospital, University Hospital of Cologne and UniReha, University Hospital of Cologne, Cologne, Germany; Department of Paediatrics, Kinderklinik München Schwabing, Technical University of Munich School of Medicine, Munich, Germany; Institute of Epidemiology and Medical Biometry, ZIBMT, Ulm University, Ulm, Germany; Children’s Hospital Krefeld, Krefeld, Germany; Otto-von-Guericke-University Magdeburg, Department of Paediatrics, Magdeburg, Germany; German Center for Diabetes Research (DZD), Munich-Neuherberg, Germany

**Keywords:** congenital adrenal hyperplasia, congenital primary hypothyroidism, patient registry, newborn screening, quality management, benchmarking

## Abstract

Congenital primary hypothyroidism (CH) and congenital adrenal hyperplasia (CAH) are targeted by the German and Austrian newborn screening. For both diseases, there are registries for quality improvement, based on standardized observational data from long-term patient follow-up, under the auspices of the DGKED study group. By September 2021, the CH registry HypoDOK includes datasets from 23,348 visits of 1,840 patients, and the CAH registry contains datasets from 36,237 visits of 1,976 patients. Here, we report on the recruitment process, patient characteristics, and research contributions from the registries, and underline that the registries are an important tool to improve patient care and outcomes. Registries for rare conditions should thus be considered as an important public health measure and they should be adequately institutionalized and funded.

## Introduction

Newborn screening programs in Germany include screening for the endocrinopathies congenital primary hypothyroidism (CH) and congenital adrenal hyperplasia (CAH).

As the most frequent neonatal endocrine disorder, CH occurs at a frequency of 1:2,000 to 1:3,000 in Europe [1]. Elevated TSH concentrations in the neonatal screening raise a suspicion of CH; TSH and fT4 concentrations in venous blood samples are determined to confirm the disease. Levothyroxine (LT_4_) substitution is initiated after confirmation of the diagnosis, so that the symptoms of untreated CH – including cognitive impairment with neurodevelopmental delay and growth retardation – can be prevented. In the pre-screening era, up to 28 % of individuals with clinically detected hypothyroidism had an IQ of <70 [2]. Although screening by TSH alone will not detect rare cases of congenital secondary hypothyroidism, occurring at a frequency of 1:16,000 to 1:100,000 [3], newborn screening for CH is a highly effective approach to prevent intellectual disability by detecting affected individuals at an asymptomatic stage [4].

CAH refers to a group of rare autosomal recessively inherited defects of adrenal cortisol and aldosterone biosynthesis, mostly caused by 21-hydroxylase deficiency due to *CYP21A2* gene mutations. In 75 % of patients with 21-hydroxylase deficiency, mineralocorticoid synthesis is also defective, putting affected individuals at risk of potentially fatal salt-wasting crises and accounting for the high mortality in CAH [5], [6]. CAH without salt-wasting is referred to as “simple virilizing” CAH. If left untreated, CAH may lead to pseudo-precocious puberty, premature closure of the epiphyses, and short stature [7]. Newborn screening for CAH was introduced in Germany in 1999 and in Austria in 2001; it aims at detecting affected individuals by elevation of 17-hydroxyprogesterone (17-OHP) levels in order to avoid adrenal crisis by early initiation of treatment, which consists of lifelong substitution with glucocorticoids and – in patients with salt-wasting CAH – additionally with mineralocorticoids [6]. Adequate treatment of CAH avoids androgen excess in affected individuals.

In Germany, there is no registry addressing all screening diagnoses, but patient registries exist for some target diseases of the newborn screening program, e. g., for cystic fibrosis [8], CH, and CAH. Here, we report on the recruitment process, patient characteristics, and research contributions of the German and Austrian national registries for CH and CAH and assess their contribution to a long-lasting high-quality care of individuals detected in newborn screening programs.

**Figure 1:**
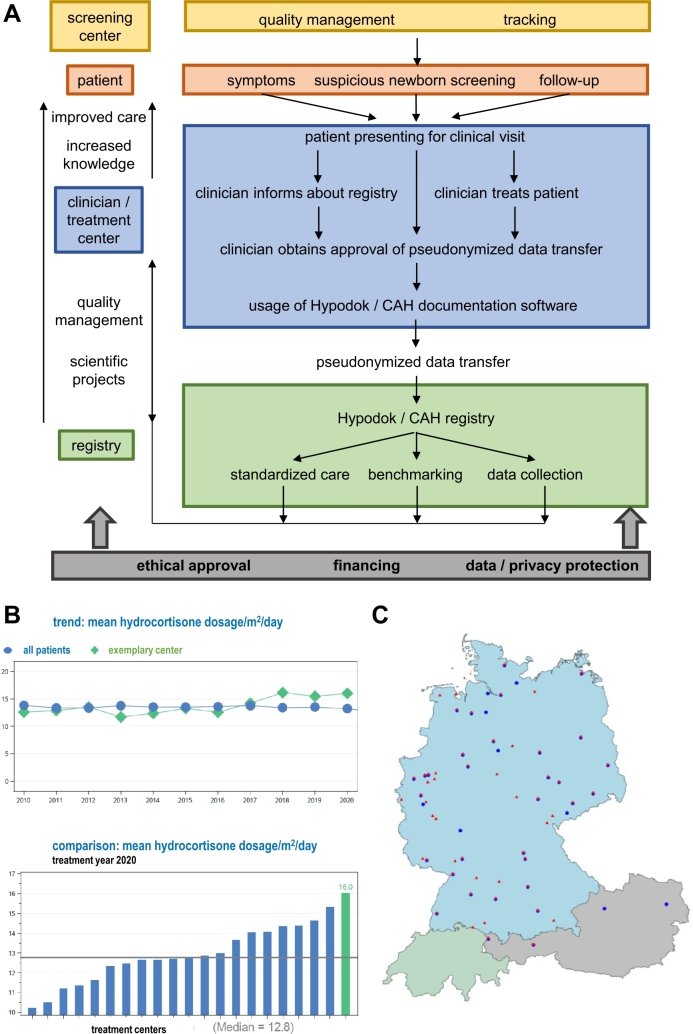
A. Dataflow from newborn screening centers and clinical treatment providers to the registry is illustrated. Roles and tasks at different levels are indicated, and appropriate funding and legal regulations are pointed out as common basis. B. Exemplary benchmarking reports for hydrocortisone dosage, as a trend of the respective center over the last years (top panel) and as a comparison between the respective center and the other participating centers (bottom panel). C. Map of centers contributing data to the CH (red triangles) and CAH (blue dots) registries.

**Table 1:** Representation of quality indicators in the benchmarking and characteristics of patients. Median and first and third quartiles are indicated for continuous variables, percentages are indicated for categorical variables. For a CAH patient, arterial hypertension was documented if the median of all registered measurements in this patient was elevated.

DGKED registry for CH: Hypo-DOK		DGKED registry for CAH	
	
Representation of quality indicators in benchmarking			
early start of therapy	age at treatment initiation (d), time lag from screening to treatment	adequate hydrocortisone dosing	dosage of hydrocortisone
confirmation of CH by low fT4/T4 concentrations	initial fT4/T4 concentrations, # of patients with low/high TSH	adequate follow-up	number of annual visits per patients
exclusion of transient forms of CH	performance of and age at withdrawal trials	monitoring growth	difference between final height and target height
adequate follow-up	number of annual visits per patients	monitoring growth	initial and most recent weight, height, and BMI SDS
monitoring growth	initial and most recent weight, height, and BMI SDS	monitoring puberty	age at menarche, difference between bone age and chronological age
monitoring psychomotor development	information on performed and necessary IQ tests	presence of arterial hypertension	proportion of patients with arterial hypertension

Patient characteristics					

Variable	*n*		Variable	*n*	
**whole cohort**			**whole cohort**		
	
sex (% male)	1,840	36.6	sex (% male)	1,975	44.1
age (yrs)	1,840	6.2 (2.3; 12.5)	age (yrs)	1,976	13.9 (7.4; 18.1)
height (cm)	1,815	92.4 (75.7; 119.0)	height (cm)	1,948	134.4 (103.3; 155.0)
height SDS	1,815	−0.04 (−0.72; 0.62)	height SDS	1,948	−0.12 (−0.99; 0.65)
weight (kg)	1,821	14.0 (9.3; 22.8)	weight (kg)	1,951	34.5 (17.2; 52.8)
weight SDS	1,821	0.23 (−0.48; 0.88)	weight SDS	1,951	0.60 (−0.27; 1.40)
BMI	1,812	16.4 (15.3; 17.7)	BMI	1,948	18.7 (16.6; 22.3)
BMI SDS	1,812	0.38 (−0.24; 0.99)	BMI SDS	1,948	0.78 (0.08; 1.55)
number of visits	1,840	10 (3; 18)	number of visits	1,976	13 (5; 27)
IQ	210	103 (94; 110)	genetic analysis performed (%)	1,765	86.2
			
**patients aged < 1 year**			salt-wasting CAH (%)	1,931	59.2
			
age at first visit (d)	1,396	11 (6; 32)	CAH without salt-wasting (%)	1,931	15.4
age at treatment initiation (d)	1,277	7 (5; 11)	non-classic CAH (%)	1,931	11.1
lag screening-treatment (d)	1,045	3 (1; 6)	heterozygous CAH	1,931	3.3
initial serum T3 (nmol/L)	683	2.1 (1.3; 2.8)	Prader stage >0 in females (%)	679	91.2
initial serum T4 (nmol/L)	624	204 (116; 1,167)	arterial hypertension (%)	1,762	13.1
initial serum TSH (mU/L)	1,827	25.6 (2.7; 150.0)	mineral corticoid treatment (%)	1,931	69.9
initial serum fT4 (pmol/L)	1,726	24.7 (17.0; 53.0)	blood pressure measured (%)	1,931	89.1
			
**patients aged > 1 year (most recent data)**			genital surgery performed (%)	1,202	40.8
			
serum T3 (nmol/L)	487	2.1 (1.6; 2.5)	hydrocortisone dosage (mg/m^2^)	1,741	14.3 (12.0; 16.8)
			
serum T4 (nmol/L)	443	166 (131; 1,471)	**patients aged ≤ 4 weeks**		
			
serum TSH (mU/L)	1,581	3.1 (1.2; 6.6)	age at first visit (d)	440	7 (0; 17)
serum fT4 (pmol/L)	1,509	20.5 (16.9; 27.4)	hydrocortisone dosage (mg/m^2^)	206	19.6 (16.5; 23.9)
IQ test necessary	1,598	73.6	screening performed (%)	440	45.5
IQ test done (age ≥ 4 yrs)	1,575	32.6	NaCl supplementation (%)	440	15.9
			
**patients aged ≥ 2 years**			mineral corticoid treatment (%)	440	48.9
	
withdrawal from trial (%)	1,840	18.9	**patients aged ≥ 2 years**		
			
age at withdrawal from trial (yrs)	348	3.2 (2.4; 5.2)	difference between chronological age and bone age (yrs)	1,476	−0.53 (−2.01; 0.48)
			
diagnosis confirmed (%)	348	63.8	**patients aged ≥ 16 years**		
			
			difference between final height and target height (cm)	729	−2.2 (−7.3; 3.3)
			age at menarche (yrs)	242	12.9 (11.7; 14.2)

## Characterization of the CH and CAH registries

### History and patient recruiting

The German national registries for CH and CAH, the “HypoDOK” registry for CH and the CAH registry, were initiated by the Study Group on Quality Management in Paediatric Endocrinology (Arbeitsgemeinschaft für Qualitätssicherung in der Pädiatrischen Endokrinologie [AQUAPE]) in 1997. In the meantime, the quality improvement group is integrated in the German Society for Paediatric Endocrinology and Diabetology (Deutsche Gesellschaft für Kinderendokrinologie und Diabetologie [DGKED]) [9], [10], [11], which continues to take responsibility for the registries.

The CH and CAH national registries allow prospective documentation of clinical follow-up data. Both registries are based on electronic health record software used at the participating centers for standardized documentation of routine clinical visits. The software packages were developed at the Institute for Epidemiology and Medical Biometry at Ulm University and can be downloaded free of charge by all interested centers in German-speaking countries (www.peda-qs.de) [12]. A center-specific signature file ensures correct location of documented patients.

Patient data collected during routine clinical visits are recorded electronically. For data transfer from clinical information systems used in hospitals – independently of the type of software that is used – there is a Health Level 7 interface, while data exchange between the administrative software in private practices and the registries is carried out with a BDT interface. Datasets contain information on phenotype, genotype, relevant laboratory results, medication, anthropometric measures, and surgical interventions. Documentation parameters were selected based on current treatment guidelines and clinical expertise of participating centers, with several group meetings to decide on parameter selection. Twice a year, pseudonymized data are transferred to the Institute of Epidemiology and Medical Biometry at Ulm University, Ulm, Germany, for central analysis, where data are validated, reported back to the treatment center for correction if necessary, and used to generate benchmarking reports on the quality of care for each individual center (Figure 1).

In both registries, the datasets of September 2021 were used as a basis for analyses.

### Benchmarking reports as a means for quality improvement

As a means of quality assurance, benchmarking reports are provided to the participating centers semi-annually. These reports contain data not only on the number of patients and visits in the respective time period and treatment center, on anthropometric follow-up measurements, and on laboratory results, but also on completeness of documentation. Important quality indicators are reported, e. g., monitoring of growth and puberty, dosing of medication, psychomotor development, and the number of follow-up visits in both CH and CAH (Table 1). Clinical centers are given feedback on their individual performance related to guideline recommendations. Outcomes are compared not only to results at the same institution in previous years, but also to data from other participating centers. Therefore, continuous benchmarking serves as a tool for internal and external quality management (Figure 1).

Here, we describe how benchmarking and documentation of clinical information can substantially contribute to quality improvement.

### The CAH registry: Patient characteristics and clinical information

**Figure 2:**
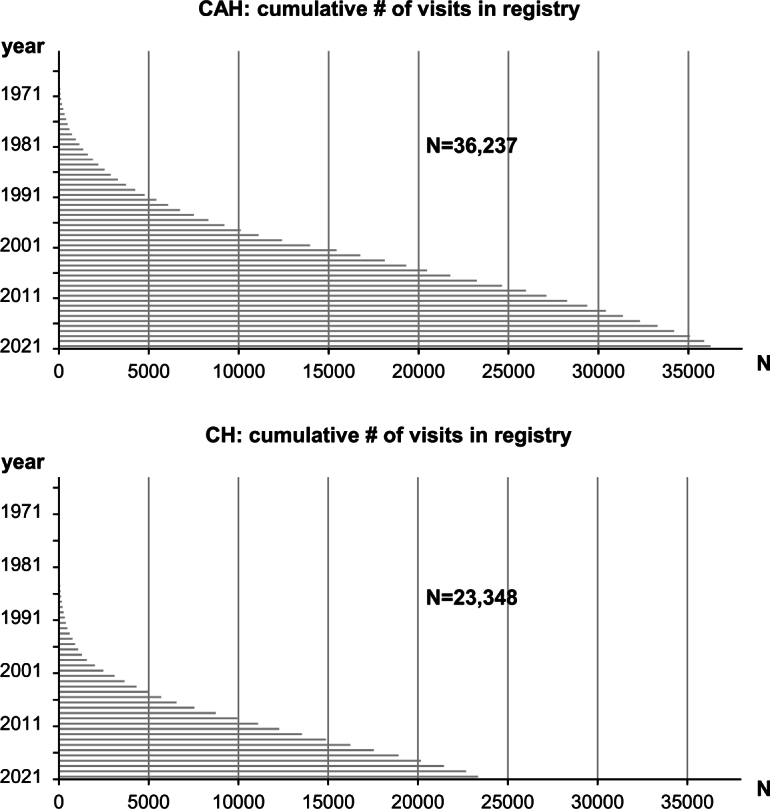
Cumulative number of visits included in the CH and CAH registries is depicted.

In total, 53 centers – 49 German and four Austrian – contributed to the CAH registry, which included datasets from 36,237 visits in 1,976 patients (Figures 1 and 2, Table 1). Datasets of a median of 13 visits per patient were included in the registry, dating back to 1960. Of the patients, 44.1 % were male, and their median age at the most recent visit was 13.9 years. Moreover, 59.2 % of the individuals suffered from salt-wasting CAH, 15.4 % suffered from classic CAH without salt-wasting, non-classic CAH was diagnosed in 11.1 % of the patients, and for 3.3 % of the patients heterozygous CAH was reported. In 679 females, the Prader stage was documented; in 91.2 % of them, it was classified as >0, indicating that some virilization was present. For 1,202 individuals, it was documented whether genital surgery had been performed or not; for 40.8 % of these individuals, genital surgery was documented (Table 1). For 1,765 patients, the information whether molecular genetic testing had been performed was present; in 86.2 % of these 1,765 individuals, molecular genetic analysis had been performed. Molecular genetic results were available for 2,149 alleles (Table 2; [13], [14]). *CYP21A2* deletions, an intronic mutation leading to aberrant splicing, and several missense mutations were among the most frequent *CYP21A2* variants reported (Table 2).

**Table 2:** (a) Affected genes in patients reported in the CH registry. (b) CYP21A2-associated variants reported most frequently in patients in the CAH registry.

(a) CH registry: affected genes in CH patients reported in the registry		

affected gene	# of patients	% of patients in registry (n=1,840)
*DUOX2*	8	0.43
*DUOXA2*	1	0.05
*NKX2-1* (TTF1)	2	0.11
*PAX8*	6	0.33
*PDS* (pendrin)	4	0.22
*TPO*	24	1.30
*TSHR* (TSH receptor)	4	0.22
*TG*	3	0.16
no variant found	19	1.03
not determined	1,769	96.14

(b) *CYP21A2*-associated variants reported most frequently in patients in the CAH registry					

*CYP21A2*-associated variant	previously also reported as	protein effect [13], [14]	# of alleles	% of analyzed alleles (n=2,149)	% of all alleles (n=1,976×2=3,952)
deletion			544	25.31	13.77
c.293-13A/C>G	I2G, 656A/C>G	aberrant splicing	506	23.55	12.80
c.518T>A	I172N	p.Ile173Asn	238	11.07	6.02
c.844G>T	V281L	p.Val282Leu	184	8.56	4.66
c.955C>T	Q318X	p.Gln319*	86	4.00	2.18
c.1069C>T	R356W	p.Arg357Trp	61	2.84	1.54
c.92C>T	P30L	p.Pro31Leu	51	2.37	1.29
c.332_339del	707-714 del, G110_8bp	p.Gly111Valfs*21	48	2.23	1.21
c.1360C>T	P453S	p.Pro454Ser	25	1.16	0.63
conversion			21	0.98	0.53
		
not determined			1,803		45.62

Based on the data in the registry, genotype–phenotype correlations in individuals with CAH were analyzed in detail in a recent study [15]. In this study, *CYP21A2* mutations were classified as null, A, B, C, or D based on residual 21-hydroxylase activity. In compound heterozygous individuals with CAH, the genotype is determined by the mutation allowing the highest residual function. In general, there was a strong genotype–phenotype correlation for mutations predicting no or only marginal residual 21-hydroxylase activity: in patients with a genotype predicting no or only 0–1 % residual 21-hydroxylase activity, salt-wasting occurred in 97 % and 91 % of the patients, respectively. For less severe genotypes, this correlation was weaker [15]. Of note, screened patients were compared to unscreened subjects. In screened patients, concordance of mild genotypes with the predicted phenotype was even lower in screened patients than in those of the pre-screening era [15].

### Content of CAH benchmarking reports and contribution to quality improvement

Besides data on the number of patients and visits in the respective time period and treatment center, the benchmarking reports focus on specific clinical aspects related to CAH. Normal growth and weight are important goals for CAH treatment. Completeness of documentation is thus essential for monitoring treatment efficiency – this information is returned to participating centers biannually. In the CAH registry, anthropometric data on height and weight were available for 1,948 patients, and thus for almost all individuals in the registry. For each patient, the individual median height standard deviation score (SDS) of all visits (or all visits in a defined time period) was determined. For the whole cohort, the median height SDS was −0.12, the median weight SDS was 0.60, and the median BMI SDS was 0.78 (Table 1). Data from the “Arbeitsgemeinschaft Adipositas im Kindes- und Jugendalter” (AGA; Working Group on Obesity in Childhood and Adolescence) were used as a reference in both the CAH and the CH registry [16]. The difference between median target height (adjusted mid-parental height) and median individual height is also included in the registry and in the benchmarking reports; in the patient cohort from the CAH registry, median target heights were 2.2 cm above median individual heights (the maximum height for each patient aged >16 years was considered). When monitoring CAH patients, special attention should be paid to acceleration of bone age and puberty, so that information on these parameters is included in the benchmarking reports. In the CAH patient registry, the median difference between chronological age and bone age was −0.53 years; the median bone age was thus more than 6 months advanced when compared to chronological age. Median age at menarche was 12.9 years.

Since monitoring for arterial hypertension is recommended in CAH patients, availability and completeness of blood pressure measurements are important quality indicators. Blood pressure data (single time point measurements) were available for 89.1 % of all patients in the registry; arterial hypertension data were present in 13.1 % of them (Table 1).

The CAH registry also gathers information on therapeutic management, which is included in the benchmarking reports. In the cohort of registered patients, median hydrocortisone dosage was 14.3 mg/m^2^/day, and 69.9 % of the patients received mineralocorticoid treatment.

In the 440 patients for whom information on birth and the neonatal age was documented, the median age at the first documented visit was 7 days; neonatal screening had been performed in 45.5 % of them – this includes all patients in the registry, not only those born after introduction of newborn screening. The median daily hydrocortisone dosage in these 440 patients was 19.6 mg/m^2^ in the neonatal period, 48.9 % of the patients received mineralocorticoid treatment, and 15.9 % of them received sodium chloride supplementation (Table 1).

### The CH registry: Patient characteristics and clinical information

In total, 63 centers – two from Austria and 61 from Germany – participated in the CH registry HypoDOK, which included datasets from 23,348 visits in 1,840 patients, with a median of 10 visits per patient (Figures 1 and 2, Table 1). Of the individuals in the CH registry, 36.6 % were male, and the median age of registered patients was 6.2 years. Anthropometric data were available for more than 1,810 patients. In the most recent year of each patient, the median height SDS was −0.04, the median weight SDS was 0.23, and the median BMI SDS was 0.38 (Table 1).

Regarding genetic analyses of CH, there were only 52 individuals in the CH registry for whom a defined molecular genetic result was available. Among these, 24 individuals carried a *TPO* gene variant (Table 2).

### Content of CH benchmarking reports and contribution to quality improvement

Early treatment initiation represents an important indicator for quality assurance in CH. In neonates included in the CH registry, treatment was initiated at a median age of 7 days, and the median interval from the positive screening result to treatment initiation was 3 days. Initial serum TSH values were available for 1,827 infants, and the initial median serum TSH was 25.6 mU/L. The initial median serum T4 value was 204 nmol/L, and the initial median serum T3 value was 2.1 nmol/L (Table 1); these values represent the first ones documented in the registry, not necessarily the first ones determined in the respective patients.

Patients with suspected CH in newborn screening programs may not always require lifelong treatment. Data on withdrawal trials of L-thyroxine supplementation to exclude transitory CH can also be deducted from the CH registry: probatory cessation of therapy was performed in 18.9 % of 1,840 patients at a median age of 3.2 years. The CH diagnosis was confirmed in 63.8 % of them (Table 1).

Normal cognitive development is an important goal of CH treatment. The latest European consensus guidelines recommend periodical evaluation of psychomotor development and school progression in all children with CH and additional workup in children with speech delay, attention deficits, and memory or behavioral problems [4]. The national German guidelines for the management of CH, which are currently under review, recommend that psychomotor development of affected children should be monitored during the first two years of therapy, and before start of school [9], [10]. The latest datasets from patients aged more than 4 years in the CH HypoDOK registry comprise IQ test results from 210 patients only. The median IQ of 103 patients was within the normal range (Table 1), with 10.5 % of the patients exhibiting an IQ of below 85 and 4.8 % of below 70.

### Proportion of new patients in the registries and newborn screening programs

To determine how many of the patients with CH and CAH detected in newborn screening are documented in the registries, the respective numbers were compared. In the years 2004–2019, 3,419 individuals with CH were detected in the German newborn screening program, with 163–279 patients per year [17]. Between 2015 and 2019, 147 patients in total were identified in the Austrian newborn screening program [18]. A recent analysis showed that in the years 2004–2017, the CH registry HypoDOK covers an average of 17.5 % of the patients with confirmed CH detected in the German newborn screening program, and in the years 2015–2019, it covers an average of 10.3 % of the patients with confirmed CH found in the Austrian newborn screening program [10].

**Table 3:** CAH patients detected in newborn screening programs in Germany (D) and Austria (A) in the last years and patients in the registry born in each of the years are indicated to determine the coverage of screened patients in the registry. n/a: numbers not available [17], [18].

	Congenital adrenal hyperplasia					

Year	# of confirmed cases in screening		# of screened patients in registry born in respective year		fraction patients in registry / patients in screening (%)	
			
	D	A	D	A	D	A
**2019**	46	5	12	0	26.1	0.0
**2018**	58	7	14	1	24.1	14.3
**2017**	48	6	16	1	33.3	16.7
**2016**	54	7	19	3	35.2	42.9
**2015**	36	9	14	2	38.9	22.2
**2014**	45	n/a	19	1	42.2	n/a
**2013**	47	n/a	15	1	31.9	n/a
**2012**	48	n/a	14	2	29.2	n/a
**2011**	44	n/a	12	4	27.3	n/a
**2010**	39	n/a	23	3	59.0	n/a
**2009**	38	n/a	22	4	57.9	n/a
**2008**	43	n/a	24	2	55.8	n/a
**2007**	57	n/a	18	6	31.6	n/a
**2006**	57	n/a	23	0	40.4	n/a
**2005**	59	n/a	26	2	44.1	n/a
**2004**	62	n/a	14	4	22.6	n/a
**All**	**781**	**34**	**285**	**36**		
**D: years 2004–2019**					**36.5**	
**A: years 2015–2019**						**20.6**

With regard to CAH, 36–62 patients with CAH per year were detected in the newborn screening program in Germany in the years 2004–2019; a total of 34 CAH patients were detected in the Austrian newborn screening program between 2015 and 2019 (Table 3) [17], [18]. The CAH registry includes data from 285 German patients who have been screened and were born in the years 2004–2019; for Austria, 7 patients who had undergone screening and were born between 2015 and 2019 are documented in the CAH registry. Thus, the registry includes data from 20.6 % of the Austrian patients and 36.5 % of the German patients detected in the newborn screening programs.

### Selected research work resulting from the CAH and CH registries

Recently, several research analyses based on data from the CAH and CH registries have been published. Besides the study that assessed genotype–phenotype correlation in individuals with CAH [15], a detailed analysis of hydrocortisone dosage was based on the data in the CAH registry [11]. Further studies analyzed the frequency of sodium chloride supplementation in patients with salt-wasting CAH [19] and the prevalence of arterial hypertension in pediatric CAH patients [20]. Recent studies from the HypoDOK CH registry elucidate the long-term follow-up and outcome of patients with CH [9], help to identify predictors of transient CH [21], or addressed guideline adherence in patients with CH before and after implementation of the first guideline for management of CH [10].

## Discussion

Both the CH and the CAH registry include data from a large number of patients treated at participating expert centers, containing real-life data on anthropometric data, laboratory results, treatment, and long-term outcome. The DGKED registries for CAH and CH also help to give direct feedback to participating centers by issuing benchmarking reports, in which quality indicators and performance of the respective center are described. Items documented in the registries and aggregated in the benchmarking reports are chosen according to current treatment guidelines and recommendations, in order to represent an important tool of quality improvement.

Moreover, the registries allow research studies based on real-life data, often with direct clinical implications. For example, a study from the CAH registry may help to identify patients at increased risk of developing arterial hypertension and to install close monitoring to avoid or treat this complication [20]. Another analysis based on the CAH registry showed that in a relevant number of patients, the daily hydrocortisone dose is above the recommendation, especially in the first months of life [11], corresponding well with results from the international, but smaller I-CAH registry [22]. In an analysis on genotype–phenotype correlations in CAH based on the German national registry, assigned phenotypes were more severe than expected in milder genotypes and in screened patients [15], which should be considered in future classification of CAH forms in order to counsel patients adequately and in order to avoid overtreatment.

Besides an increased socio-economic burden, overtreatment of CH has individual disadvantages for affected patients and their families. Whereas lower TSH thresholds in newborn screening programs for CH will lead to detection of more patients with mild or transient forms of CH, robust evidence for improved psychomotor development after treatment of mildly affected children is still scarce [23]. Distinction between transient and permanent forms of congenital hypothyroidism is therefore crucial, and several recent studies deal with this issue [24], [25], [26]. In an analysis based on the CH HypoDOK registry, the screening TSH value and L-T4 dosage at 1 and 2 years were identified as predictors of transient hypothyroidism [21], illustrating how long-term follow-up of patients detected in newborn screening can have an impact on future improvement of screening programs, e. g., by determining cutoff values. Special attention needs to be paid to preterm infants with a very low birth weight, in whom CH may be missed due to a delayed rise of TSH values [27].

So far, the national registries for CH and CAH do not cover all patients with these conditions that were detected in newborn screening programs, especially in CH; patients in the registries are mostly enrolled by pediatric endocrinology specialists who are aware of the quality initiative of the DGKED and the software used for standardized data collection. Thus, there is a selection bias towards patients treated in expert centers – however especially CH patients are also followed by general private pediatricians. Extending the registries’ range by addressing more pediatricians in smaller centers may help to increase visibility of the registries and thus lead to a higher patient coverage and to an increase of data validity, although from a perspective of guideline-oriented patient care regular surveillance of both endocrine disorders by pediatric endocrinologists seems preferable. Participation in the benchmarking process as a means of quality management may be one incentive to participate in the registry. Regional networks of accredited centers and primary care pediatricians or an obligation for standardized documentation in specialist centers represent other strategies to improve the registries’ coverage [10].

Linking up screening laboratories or centers with specialist care may also improve the registries’ range, which may ultimately contribute to a sustainable effect of newborn screening programs as a public health measure. In Germany, systematic follow-up of neonates with positive screening results is not required and depends on the federal states. In Bavaria, the “Landesamt für Gesundheit und Lebensmittelsicherheit” performs institutionalized follow-up of positive screening results; in other federal states, this is often performed by the screening laboratories and depends on local resources rather than on continuous funding [28]. Not only tracking of positive screening results, but also long-term follow up of patients detected in newborn screening programs in Germany lacks institutionalization and continuous funding. However, reliable information on long-term follow-up of patients detected in newborn screening programs is essential to assess the benefit of newborn screening programs and identify gaps and suboptimal care. Inclusion of more detailed information on the molecular genetic background of CH and CAH in the respective registries may help to understand genotype–phenotype correlations, and may thus further increase knowledge on these rare endocrine conditions.

Therefore, future endeavors for rare disease registries will have to aim at building high-quality databases assembling observational clinical information. Meeting ethical and legal requirements, notably data protection regulations, are crucial tasks for registry holders [29], and will require reliable funding.

Internationally, CAH is not generally included in newborn screening programs. Besides infant deaths due to salt-wasting, considerable morbidity from CAH arises from hyperandrogenemia in older affected individuals. In order to assess the benefit of a screening program for CAH in asymptomatic newborns, a systematic evaluation of the long-term outcome of patients is important [30]. Optimal care of individuals with rare diseases should aim at a reduction of morbidity and mortality and at improved quality of life of affected individuals. Lifelong surveillance of patients with rare diseases identified in newborn screening programs and pooling of data in registries may facilitate research activities as well as collaboration of specialists involved in patient care. By developing clinical benchmarks for participating centers, registries may also play an important role in quality management [31].

A way to promote standardized care of patients with rare diseases is the introduction of guidelines. A recent study on guideline adherence in the management of CH showed that formal assessment of development and hearing was documented less often than recommended [10]. Once identified, this problem may be addressed in the future, so that the registry may contribute to a sustainable effect of the newborn screening program for CH on the long-term outcome of affected individuals.

Guideline adherence in the management of rare conditions as a means of quality management represents a physician-centered approach. However, individuals affected by rare diseases may have other points of view and often focus on participation in social life and work [32]. Therefore, in the future patient-reported outcome measures should be included in the registries’ datasets [29], [33]. When individuals affected by a rare disease grow older and potentially leave their family pediatrician or pediatric endocrinologist, different aspects of counseling should be taken into account. The national registries for CH and CAH include information on children, but also on adults. One of their strengths is that they are based on a documentation software allowing introduction of new variables. Adjusting the documentation software to the changing needs of growing patients and to a changing environment is an important task for participating experts and will help to increase validity of the data included in the registries.

The importance of registries in the management of rare conditions and their role in quality assurance is increasingly being recognized, also by international bodies. With its European Reference Networks (ERNs) for different groups of rare disorders, e. g., the Endo-ERN for rare endocrine conditions, the European Commission institutionalized programs for rare diseases. One aim of these networks is to harmonize patient care and documentation across Europe [10], [31], [33].

The need for institutionalized programs for the long-term follow-up of patients detected in newborn screening and the necessity of continuous funding by the state need to be focused on in the future. In pediatric oncology, since 1980, a wide majority of patients in Germany is included in a nationwide registry gathering information on epidemiology of childhood malignancies. It is used by researchers and clinical centers to adjust treatment protocols, and it is needed for administrative means, e. g., for medical care planning [34], thus having the potential to serve as a blueprint for other registries of rare conditions in childhood.

The experience of the DGKED quality management study group demonstrates that the registries for CH and CAH are able to gather and manage observational data from long-term follow-up of many affected individuals, thus fostering exchange of knowledge and clinical experience between the centers. By providing biannual benchmarking reports for participating centers, they directly contribute to quality improvement. The resulting scientific studies have strong clinical implications, underlining that databases managing follow-up data from routine clinical practice are an important tool to improve patient care and outcome. Registries for rare conditions should thus be considered as an important public health measure and should be adequately institutionalized and funded, so that they contribute to a sustainable effect of the newborn screening program on the long-term outcome of affected individuals.

## Abbreviations

17-OHP: 17-hydroxyprogesterone
AQUAPE: Arbeitsgemeinschaft für Qualitätssicherung in der Pädiatrischen Endokrinologie (Study Group on Quality Management in Paediatric Endocrinology)
CAH: congenital adrenal hyperplasia
CH: congenital primary hypothyroidism
LT_4_: levothyroxine
